# Factors Associated with Reliable Contact Tracing During the 2021 Ebola Virus Disease Outbreak in Guinea

**DOI:** 10.1007/s44197-024-00202-y

**Published:** 2024-02-19

**Authors:** Mory Keita, Ibrahima Sory Cherif, Jonathan A. Polonsky, Samuel T. Boland, Youba Kandako, Mahamoud Sama Cherif, Mamadou Kourouma, Aly Antoine Kamano, Houssainatou Bah, Ibrahima Sory Fofana, Georges Alfred Ki-zerbo, Stephanie Dagron, Dick Chamla, Abdou Salam Gueye, Olivia Keiser

**Affiliations:** 1 World Health Organization, Regional Office for Africa, Brazzaville, Congo; 2 Institute of Global Health, Faculty of Medicine, University of Geneva, Geneva, Switzerland; 3 Country Office for Guinea, World Health Organization, Conakry, Guinea; 4 Geneva Centre of Humanitarian Studies, Faculty of Medicine, University of Geneva, Geneva, Switzerland; 5 Epicentre, Geneva, Switzerland; 6 Chatham House, London, UK; 7 Ministry of Health, Regional Health Direction of Faranah, Faranah, Guinea

**Keywords:** Ebola virus disease, Contact tracing, Surveillance, Guinea

## Abstract

**Background:**

In 2021, an Ebola virus disease (EVD) outbreak was declared in Guinea, linked to persistent virus from the 2014–2016 West Africa Epidemic. This paper analyzes factors associated with contact tracing reliability (defined as completion of a 21-day daily follow-up) during the 2021 outbreak, and transitively, provides recommendations for enhancing contact tracing reliability in future.

**Methods:**

We conducted a descriptive and analytical cross-sectional study using multivariate regression analysis of contact tracing data from 1071 EVD contacts of 23 EVD cases (16 confirmed and 7 probable).

**Results:**

Findings revealed statistically significant factors affecting contact tracing reliability. Unmarried contacts were 12.76× more likely to miss follow-up than those married (OR = 12.76; 95% CI [3.39–48.05]; *p* < 0.001). Rural-dwelling contacts had 99% lower odds of being missed during the 21-day follow-up, compared to those living in urban areas (OR = 0.01; 95% CI [0.00–0.02]; *p* < 0.01). Contacts who did not receive food donations were 3× more likely to be missed (OR = 3.09; 95% CI [1.68–5.65]; *p* < 0.001) compared to those who received them. Contacts in health areas with a single team were 8× more likely to be missed (OR = 8.16; 95% CI [5.57–11.96]; *p* < 0.01) than those in health areas with two or more teams (OR = 1.00; 95% CI [1.68–5.65]; *p* < 0.001). Unvaccinated contacts were 30.1× more likely to be missed compared to vaccinated contacts (OR = 30.1; 95% CI [5.12–176.83]; *p* < 0.001).

**Conclusion:**

Findings suggest that contact tracing reliability can be significantly influenced by various demographic and organizational factors. Considering and understanding these factors—and where possible addressing them—may be crucial when designing and implementing contact tracing strategies during future outbreaks in low-resource settings.

## Introduction

The largest, longest, and deadliest outbreak of Ebola virus disease (EVD) occurred in West Africa from December 2013 to March 2016, causing 28,652 infections and 11,325 deaths in 10 countries. Of these, 99% occurred in just three countries: Guinea, Sierra Leone, and Liberia. Seven years after the outbreak was declared over, Guinea faced a new EVD outbreak. This outbreak, near the epicenter of the previous, lasted from 14 February to 19 June 2021, and resulted in 12 known deaths [1, 2]. Evidence had previously suggested that EVD outbreaks typically begin with a single case of zoonotic transmission followed by human-to-human transmission via direct contact or contact with infected bodily fluids or contaminated fomites [3]. However, the 2021 Guinea epidemic—as well as other recent epidemics and investigations—showed that EVD outbreaks may be caused by virus persisting in and transmitting from a survivor months or, in this case, years later [4, 5].

Regardless of origin, once an outbreak has occurred, the biological features of EVD—requiring a contact with body fluids for a possibility of human-to-human transmission—make contact tracing a foundational strategy to interrupt the outbreak [3]. Contact tracing is defined by the World Health Organization (WHO) as the monitoring process through which people who have been in close contact with someone infected with a pathogen during the time when they were infectious are closely followed and observed for development of any relevant signs or symptoms indicative of disease. As applied to EVD control, contact tracing consists of the identification and listing of contacts; tracing them (i.e., locating them and establishing initial contact); and finally, daily follow-up with them for the 21-day period of EVD’s incubation period, with the core dual aims of limiting the spread of an infectious disease through isolation and offering early support and treatment to any suspected case [6]. Contact tracing was a key element in containing the 2014–2016 EVD outbreak in West Africa [7], as well as other recent outbreaks in Democratic Republic of Congo (DRC) [8, 9]and Guinea [10].

The successful interruption of EVD transmission through contact tracing—meaning systematic and complete listing and subsequent 21-day follow-up can only be ensured if it is rapidly implemented upon identification of an associated EVD case, which generally includes the time taken for laboratory confirmation [7]. The completeness of the 21-day follow-up is considered a key performance indicator for overall surveillance and containment, because it impacts the duration of the outbreak and response. The definition of contact implies that a contact is not symptomatic, as for known contacts, the presence of a single symptom suggests the person should be categorized as a suspected EVD case requiring additional public health actions, such as isolation and clinical support. Therefore, for EVD, a contact is not sick, is not systematically quarantined (though it is recommended to reduce movements and travel) and is seen by contact tracers to assess their health and promptly detect EVD symptom onset up to twice a day (according to their willingness to be followed up).

However, while contact tracing is understood to be a critical tool for containing outbreaks (and a significant amount of labor is therefore assigned to its function), critical assessments of the factors effecting its performance are limited in number and rigor. This is despite known issues in recent outbreaks. For example, a recent study estimating the completeness of contact tracing during the 2018–2020 EVD outbreak in eastern DRC (using the capture and recapture method) highlighted that contact tracing efforts performed well at identifying contacts during the listing stage, but performed poorly during the contact follow-up stage [11]. Some efforts to improve effectiveness have focused on improving contact monitoring tools by digitizing as much as possible [12], for example, but few studies have focused on other factors.

In this study, we describe and analyze contact tracing activities as conducted in urban and rural areas of the Nzerekore Health District from February to May 2021. The main objective of the study was to understand the factors associated with the reliability of 21-day follow-up among listed contacts, so as to inform and help guide contact tracing efforts in response to future outbreaks in similar contexts.

## Methods

### Setting

The study was conducted in the Nzerekore health district in the Republic of Guinea. The district covers about 3632 km^2^ and has an estimated population of 497,667 inhabitants in 2021 according to the Third General Census of Population and Housing (RGPH3) [13]. It is composed of seventeen health areas. The EVD outbreak occurred from 14 February to 19 June 2021.

### Study Design and Participants

We conducted a descriptive and analytical cross-sectional study. Participants consisted of all contacts identified by epidemiological investigation teams for confirmed and probable cases during the data collection period (February to May 2021). The definition of contact was aligned with the WHO definition: any person with no sign or EVD symptom and who had been exposed to any confirmed (alive or dead) or probable EVD patient or with their bodily fluids within the past 21 days.

Accordingly, contacts were categorized into four types: Category 1 were people who had direct contact with body fluids, such as blood, saliva, vomit, breastmilk, or sperm of an EVD-infected patient; Category 2 had directly touched the alive or dead EVD-infected patient’s body; Category 3 had touched clothes of the EVD case or had shared the same linens or utensils; and Category 4 have slept in, eaten in, or otherwise shared the same house with an EVD-infected patient [6].

The contact tracing mechanism was designed at the operational level (i.e., by the response coordination committee) and implemented at the tactical level (i.e., in the community). Local agents including community One Health platform members were identified and trained to become contact tracing-specific Community Relays (ReCo). Generally, 6 to 8 contact-tracing ReCo would work under a designated supervisor and would see 15–20 contacts daily. While all contacts had only one ReCo, some health zones had two or more supervision teams coordinating their work, depending on: the uncertainty of the initial investigation; the dispersion of contacts (i.e., the geographical grouping of contacts in a zone); the relative presence of contacts classed as high risk; and the overall number of listed and unreported contacts in a given area. As per WHO guidance regarding the incubation period for EVD [6], the follow-up period was 21 days from the day following last contact with the index case. The supervisor was responsible for listing the contacts as completely as possible; partially filling the individual contact-tracing form that would later be given to the ReCo; and summarizing the information in a form for transmission to the surveillance pillar team. This information was then supplemented with additional information, thereby constructing a contact linelist.

The contact linelist is an outbreak-specific database of identified contacts, detailing each contact’s socio-demographic information; pertinent information about the index case; and the maximum theoretical date of last exposure (for greater precaution and operational ease, generally defined as the date the index case was isolated from the community). For every day of follow-up, the contact was either ‘seen’ (that is, directly observed and interviewed to note any visible signs and symptoms), or ‘not seen’. Contacts not seen were also divided into different categories: those not seen for a day or two (‘single absence’); those not seen for three consecutive days (‘lost to follow-up’); those that moved outside of their registered health districts (were ‘displaced’); those that had never been traced; and known exposures without identification (classified as ‘unknown contacts’). A contact’s status could vary throughout the tracing period (e.g., if they were lost to follow-up, but then successfully traced). During this response, a surveillance unit (alternatively called a ‘cell’) was established with the sole focus of analyzing the characteristics of contacts who were lost to follow-up or displaced. The aim was to inform and reinforce the overall contact tracing strategy by trying to identify and trace contacts that were never seen, lost to follow-up, or displaced.

**Fig. 1:**
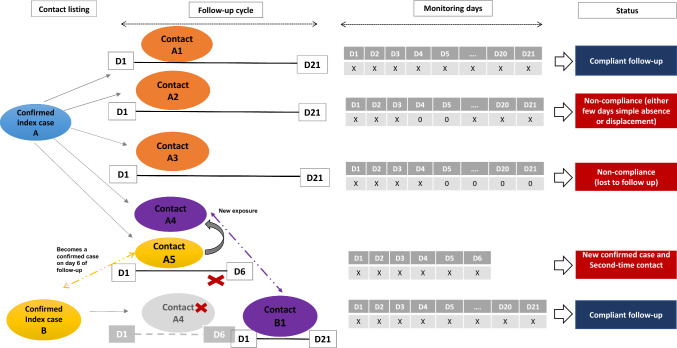
Follow-up procedure for EVD contacts and completeness criteria

During the listing process, different sources of information or an unclear or unfinished case investigation occasionally led to one person being listed multiple times for the same index case. In addition, some contacts may have been exposed to two different confirmed cases. For this study, such duplicates were identified and only one record kept for purposes of analysis. Where a contact already being contact traced had a new exposure to another case, they were followed for a second time—i.e., such that the contact tracing procedure recommenced based on the date of isolation of the more recent case (Fig. 1). Such contacts were then flagged as ‘recycled’, and their initial follow-up information excluded from analyses to avoid duplication.

### Data

Databases of alerts, cases (suspected, confirmed, and probable), and contacts were examined. The main indicator being sought was the ‘completeness’ of contact follow-up over the full 21-day incubation period (this being defined as the consistent physical presence and willingness to answer all questions from a surveillance team regarding the occurrence of one or more symptoms). ‘Non-completeness’ was therefore defined as any failure to attend one or more days of follow-up for any reason.

Socio-demographic variables (age, gender, occupation, residence, and marital status), as well as several key performance indicators (vaccination status; number of contact tracing teams and whether the contact was receiving food support from the response team) were included for analysis so as to assess the impact of various factors on contact tracing completeness. Additional analyses included: delay in notification of suspected cases (time between the onset of symptoms and notification to contact tracing staff); alert delay (time between the onset of symptoms and initiation of alert by contact tracer); and isolation delay (time between the onset of symptoms and isolation of suspected case).

### Analysis

STATA^®^ version 14.1 (Stata Corporation, College Station, Texas, United States of America) and R version 4.0.2 were used [14] to perform different analyses (described below). The analyses were performed without imputation of missing values, due to the very low percentage of missing data (0.01%). We used univariable and multivariable logistic regressions to analyze the association between the dependent variable and the independent variables specified above. In the multivariable analysis only univariable predictors with a *p* value ≤ 0.2 were considered and we used a bottom-up stepwise procedure to select the final variables that were retained. We calculated crude and adjusted Odds Ratio (OR) with their 95% confidence intervals (CI). Different models were compared based on the Akaike Information Criterion (AIC). Multicollinearity among predictors was checked by the variance inflation factor. The final model was validated using the coefficient of discrimination.

## Results

### Characteristics of the EVD Cases

**Fig. 2:**
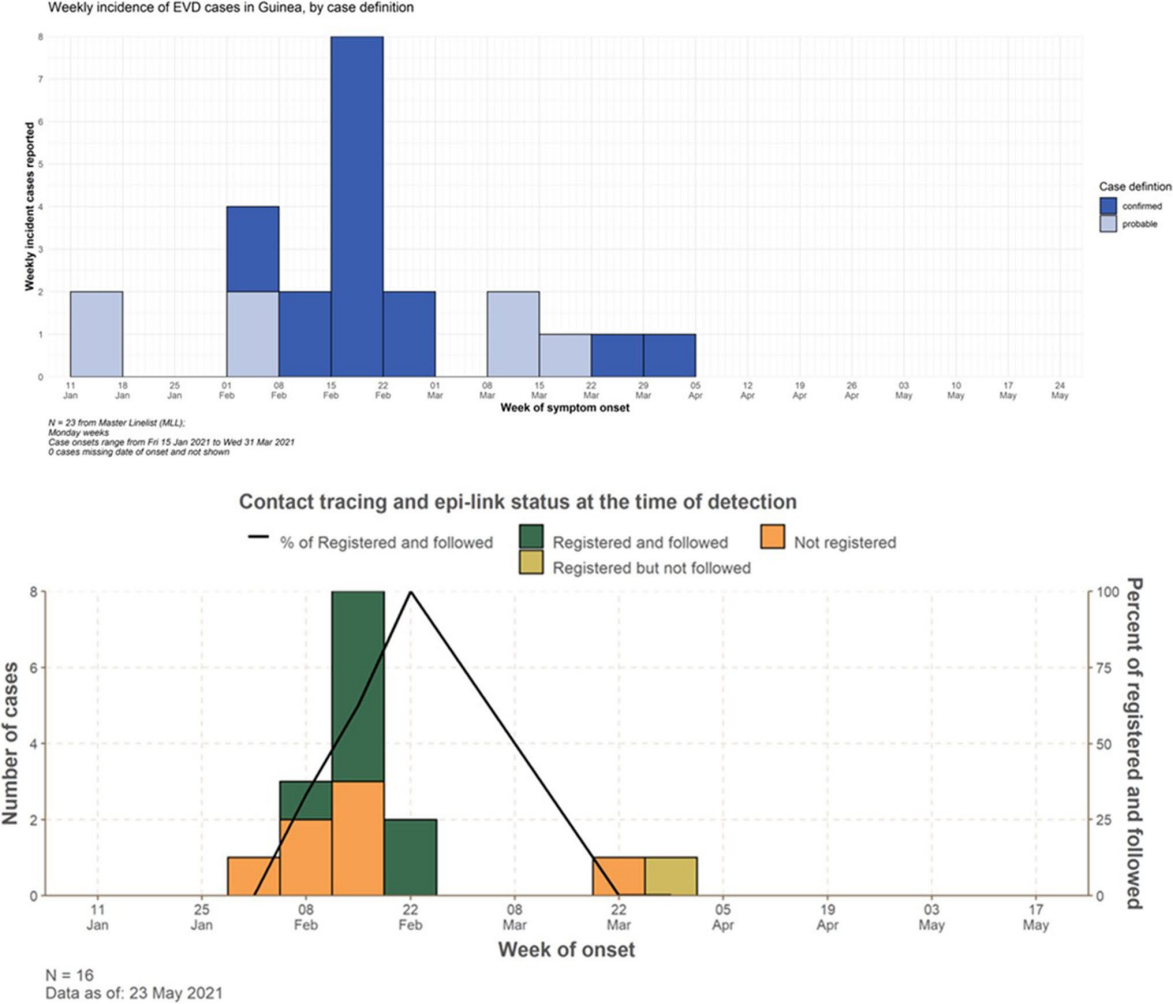
**A** Number of EVD cases (confirmed and probable) by week of symptom onset in Guinea between February and May 2021. **B** Number of confirmed EVD cases by week of onset and contact tracing status including epidemiological link (‘epi-link’) at time of detection between February and May 2021. Here, ‘epi-link’ refers to a known link with a preceding confirmed case (i.e., previously registered as a contact of another case) or not at the time of detection

**Fig. 3:**
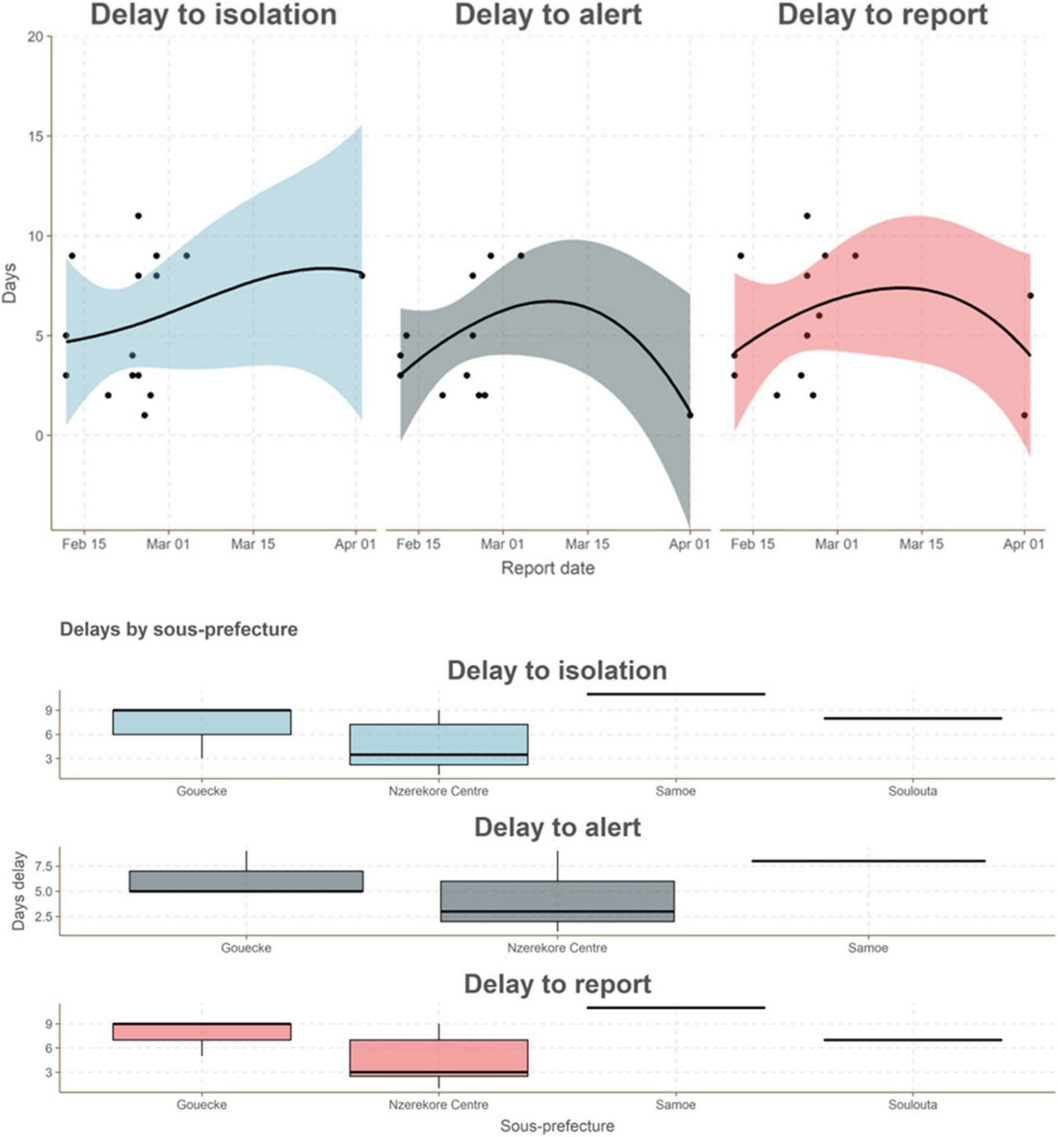
**A** Evolution of delays to isolation, to alert, and to report over time of EVD confirmed and probable cases in Guinea between February and May 2021. **B** Delays to isolation, to alert, and to report of EVD confirmed and probable cases by affected sous-prefecture in Guinea between February and May 2021

A total of 23 EVD cases (16 confirmed and 7 probable) were reported between the outbreak’s declaration on 14 February 2021 and the end of the epidemic on 19 June 2021. All cases were reported in the Prefecture of Nzerekore, with five sub-prefectures affected. Initial investigations revealed that 8/16 (50%) of confirmed cases were known and followed as contacts at time of detection. Further investigation indicated that seven confirmed cases (43.8%) were epidemiologically linked but were not recorded as contacts prior their confirmation. One confirmed case was recorded as a contact but was not followed up by contacts tracing teams (Fig. 2). For confirmed cases, the delay to report varied between 1 and 11 days; the delay to alert between 1 and 9 days; and the delay to isolation between 1 and 11 (Fig. 3).

### Characteristics of the EVD Contacts

The overall contact follow-up rate was 95%, while the overall completeness rate was 52.8% (these numbers are not identical as contacts could be followed up without 100% success, as previously described).

Of the 1071 contacts analyzed, 688 (64.2%) were aged 25 years and above; 510 (47.62%) were male and 561 female (52.6%); 706 (66.9%) were married; 450 (42.6%) were health workers; 786 (73.4%) were residing in urban areas; and 573 (54.3%) were high school graduates. A total of 905 (85.8%) received food aid, and 1030 (97.63%) were vaccinated against EVD (Table 1).

**Table 1:** Characteristics of EVD contacts in Nzerekore in the 2021 outbreak

Independent variables	Count	Percentage
Age groups (years)	*N* = 1071	
0–24 years old	383	35.76
25 years and over	688	64.24
Sex	*N* = 1071	
Male	510	47.62
Female	561	52.38
Marital status	*N* = 1055	
Married	706	66.92
Widowed/divorced	38	3.60
Unmarried	311	29.48
Educational level	*N* = 1055	
Unschooled	231	21.90
Primary level	155	14.69
Secondary level	573	54.31
Higher level	96	9.10
Contact occupations	*N* = 1055	
Doctors	27	2.56
Nurses/midwives/students	423	40.09
Merchants	57	5.40
Cultivators	108	10.24
Housewives	293	27.77
Other non-health public professionals	147	13.93
Residence of contact	*N* = 1071	
Urban	786	73.39
Rural	285	26.61
Household receiving food donations	*N* = 1055	
Yes	905	85.78
No	150	14.22
Number of assigned contact tracing teams	*N* = 1055	
One	380	36.02
Two or more	675	63.98
Vaccination status	*N* = 1055	
Vaccinated	1030	97.63
Unvaccinated	25	2.37

### Univariable Analysis of Factors Associated with Non-completeness Contact Tracing

In the univariable analysis, multiple factors had statistically significant associations with EVD contact tracing completeness. This included: age, education, and employment in certain occupations. It also included whether a contact lived in an urban or rural area; and whether or not the area had more than one contact tracing team. Data for each is presented in turn.

Odds indicates that contacts in the 0–24 age group were 1.6× more likely to be missed with follow-up than those above 25 years of age (OR = 1.62; 95% CI [1.27–2.06]; *p* < 0.001). Unmarried contacts were 4.27× more likely to miss follow-up than married contacts (OR = 4.27; 95% CI [1.56–11.67]; *p* < 0.01). Contacts with no education or with primary or secondary education were 3.5, 2.9, and 2.3× (respectively) more likely to discontinue follow-up than those with tertiary education (OR = 3.58; 2.94; 2.30; 95% CI [2.13–6.03]; [1.70–5.10]; [1.42–3.71]; *p* < 0.001; *p* < 0.001; *p* < 0.001). Contacts with certain occupations were also more likely to miss follow-up. This included those with occupations, such as farmer, housekeeper, laborer, merchant, and nurse/midwife, who were 7.4, 6.6, 6.3, 5.5, and 4.0× (respectively) more likely to miss follow-up than doctors (OR = 7.46; 6.63; 6.32; 5.55; 4.09; 95% CI [2.41–23.05]; [2.23–19.67]; [2.08–19.19]; [1.70–18.10]; [1.39–12.05]; *p* = 0.01; *p* < 0.01; *p* < 0.001; *p* < 0.01; *p* < 0.01). Those contacts who lived in rural areas had 93% lower odds of missing the 21-day follow-up, compared to those living in an urban community (OR = 0.07; 95% CI [0.04–0.10]; *p* < 0.001). In areas with a single follow-up team, contacts were 1.7× more likely to miss follow-up compared to areas with two or more teams (OR = 1.70; 95% CI [1.39–2.32]; *p* < 0.01). Unvaccinated contacts were 13.8× more likely to be missed compared to those vaccinated (OR = 13.8; 95% CI [4.19–45.54]; *p* < 0. 001) (Table 2).

**Table 2:** Factors associated with non-completeness of contact tracing in Nzerekore during the 2021EVD outbreak, using univariable analysis

Independent variables	Odds ratio (OR)	95% CI	*p* value
Age groups (years)			
25 years and over (ref)	1		
0–24 years old	1.62	[1.27–2.06]	0.000***
Sex			
Male (ref)	1		
Female	0.78	[0.61–1.00]	0.05*
Marital status			
Married	1		
Widowed/divorced	3.15	[0.36–26.87]	0.29
Unmarried	4.27	[1.56–11.67]	0.00***
Educational level			
Superior (ref)	1		
Secondary	2.30	[1.42–3.71]	0.00***
Primary	2.94	[1.70–5.10]	0.000***
Unschooled	3.58	[2.13–6.03]	0.000***
Contact occupations			
Doctors (ref)	1		
Nurses/midwives/students	4.09	[1.39–12.05]	0.01*
Merchants	5.55	[1.70–18.10]	0.00***
Cultivators	7.46	[2.41–23.05]	0.000***
Housewives	6.63	[2.23–19.67]	0.00**
Other non-health public professionals	6.32	[2.08–19.19]	0.00***
Residence of contact			
Urban (ref)	1		
Rural	0.07	[0.04–0.10]	0.000***
Household receiving food donations			
Yes (ref)	1		
No	1.47	[1.04–2.09]	0.02**
Number of contact tracing teams			
Two or more (ref)	1		
One	1.70	[1.39–2.32]	0.00***
Vaccination status			
Vaccinated	1		
Unvaccinated	13,8	[4.19–45.54]	0.00***

*p ≤ 0.05, **p ≤ 0.01, ***p ≤ 0.001

### Multivariable Analysis of Factors Associated with Non-completeness Contact Tracing

As in the univariable analysis, in the multivariable analysis, multiple factors were significantly associated with EVD contact tracing completeness. This included: age, gender, whether contacts lived in a rural or urban community, whether contacts received food donations, and whether or not the area had more than one contact tracing team. Data for each is presented in turn.

Odds also showed that contacts in the 0–24 age group were 2.3× more likely to miss follow-up than those in the 25 + age group (OR = 2.38; 95% CI [1.70–3.34]; *p* < 0.001). Female contacts had 22% lower odds of missing the 21-day follow-up (OR = 0.78; 95% CI [0.51–0.98]; *p* = 0.03) compared with men. Unmarried contacts were 12.76× more likely to miss follow-up than those married (OR = 12.76; 95% CI [3.39–48.05]; *p* < 0.001). Contacts living in rural area had 99% lower odds of missing the 21-day follow-up compared to those living in urban area (OR = 0.01; 95% CI [0.00–0.02]; *p* < 0.01). Contacts who did not receive food donations during the follow-up period were 3× more likely to be missed (OR = 3.09; 95% CI [1.68–5.65]; *p* < 0.001) compared to those who received food donations. Contacts in health areas with a single team were 8× more likely to miss follow-up (OR = 8.16; 95% CI [5.57–11.96]; *p* < 0.01) than those in health areas with two or more teams (OR = 1.00; 95% CI [1.68–5.65]; *p* < 0.001). Unvaccinated contacts were 30.1× more likely to be missed compared to those vaccinated (OR = 30.1; 95% CI [5.12–176.83]; *p* < 0.001) (Table 3).

**Table 3:** Factors associated with non-completeness of contact tracing in Nzerekore during the 2021 EVD outbreak, using multivariable analysis

Independent variables	Adjusted odds ratio (aOR)	95% CI	*p* value
Age groups (years)			
25 years and over (ref)	1		
0–24 years old	2.38	[1.70–3.34]	0.000***
Sex			
Male (ref)	1		
Female	0.70	[0.51–0.98]	0.03**
Marital status			
Married (ref)	1		
Widowed/divorced	2.79	[0.27–28.29]	0.38
Unmarried	12.76	[3.39–48.05]	0.000***
Residence of contact			
Urban (ref)	1		
Rural	0.01	[0.00–0.02]	0.000***
Household receiving food donations			
Yes (ref)	1		
No	3.09	[1.68–5.65]	0.000***
Number of contact tracing teams			
Two or more (ref)	1		
One	8.16	[5.57–11.96]	0.00***
Vaccination status			
Vaccinated	1		
Unvaccinated	30,1	[5.12–176.83]	0.00***

*p ≤ 0.05, **p ≤ 0.01, ***p ≤ 0.001

## Discussion

With the increasing frequency of EVD outbreaks in Africa [15] and the established role of contact tracing in containing transmission [16], there remains a need to understand the various factors that contribute to contact tracing success and failure. Indeed, to the authors’ knowledge, only one study roughly similar to this was conducted (in Port-Loko, Sierra Leone) [17].

Contact tracing completeness was a significant problem, though was certainly less so than has been documented elsewhere. The average contact follow-up rate was more successful compared with the earlier 2013–2016 West Africa EVD Epidemic in Guinea [18], Sierra Leone [19] and Liberia [20], and also compared with other recent outbreaks such as one in the Democratic Republic of Congo [8]. This is possibly the result of the post-2014 epidemic reforms undertaken by Guinea. Therefore, in the 2021 EVD outbreak in Guinea, many response staff had received training through Field Epidemiology Training Programs (FETPs) [10]. However, overall contact tracing completeness was still low (52.8%), which may explain the long delay between the onset of symptoms and notification and isolation that were identified. This has significant implications for survivability and containment [21]. In sum, despite the relatively high rate of follow-up in our study compared with other outbreaks, half of all confirmed cases were not previously known as contacts. This could have led to intense community transmission as experienced in previous outbreaks [22]. This was likely mitigated by the overall high vaccination rate among contacts (the result of earlier vaccination campaigns as well as ones rolled out in response to this outbreak) that may have contributed to the rapid interruption of the epidemic [23].

Taken together, it is clear that contact tracing is an integral part of any EVD response; is often not fully successful despite its importance; and is also poorly understood and under-discussed in the literature base. To help to address this research gap, this study investigated predictive factors for the completion of contact follow-up in the 2021 EVD epidemic in Guinea. Three factors related to the contacts themselves—and two related to the organization of the response—were associated with the relative completeness of contact tracing.

Younger contacts (i.e., those under 25 years old) were more likely to miss follow-up than older ones. This finding contrasted with what was observed by Jonathan et al. examining the 2019—2020 Kivu epidemic in DRC, who found that contacts in older age groups had a significantly higher probability of being missed than contacts in the youngest age group (defined in the study as 0–15 years) [11]. It is possible the discrepancy in examined age ranges could explain this difference, or it could be explained by cultural differences and varied prior experiences with EVD between the two countries. In Guinea, for example, youth often have a strong influence on discourse and beliefs within the community around issues such as EVD [24]. This can have a negative effect: during the earlier West Africa epidemic in Guinea, young people were primarily responsible for various attacks that occurred against response teams, and were often very resistant to public health interventions [25].

In Guinea, male contacts were also more likely to miss follow-up than female contacts. This is consistent with a study in the DRC, where male contacts had slightly (though statistically significantly) greater odds of being lost to follow-up [11]. Similarly, in Sierra Leone, being female was also associated with successful contact tracing over a 21-day follow-up period [17]. A possible theory is that women are often more routinely present at home [26]. Conversely, male contacts are generally more likely to work outside the home and therefore less likely to be physically present when tracing teams arrive at households. One alternative may be to consider, circumstantially, remote contact tracing via telephone. However, this may be ineffective, because of frequently poor network coverage; the possibility that a contact lacks the necessary equipment, phone credit, or phone charge to make and receive calls; and the possibility that a contact may develop illness without self-reporting under an unobserved contact tracing system. Another possible resolution would be for contact tracing teams to arrange visits away from the home, though this introduces logistical challenges as well as the possibility of stigmatizing a contact at their place of work and in the wider community.

Unmarried contacts were significantly more likely to miss follow-up than those who were married. This result is consistent with the findings of a study on the effect of knowledge and perceptions of risks on Ebola-preventive behaviors in Ghana, which demonstrated that the never married and divorced persons were significantly less likely to take action to avoid Ebola than those who were married [27]. Marriage’s effect on preventive health behaviors is extensively documented in the literature [28, 29]. For Ebola, it is believed married persons may be worried about the consequences for their close relatives.

Urban-dwelling contacts were more likely than rural-dwelling contacts to miss follow-up. A similar trend was observed in Sierra Leone [17]. This may be related to associations related to profession: economic, and professional activities in rural areas are often limited to a relatively defined geographical area, which may naturally limit contacts’ overall movement thereby easing contact tracing activities.

When contacts were provided with food donations (compared to when they were not), this resulted in very significant differences in contact tracing completeness. This may be particularly important in rural areas. As in many sub-Saharan African countries, Guinea is a developing country where roughly 21.8% of households are living in a food-insecure state. Rural populations are the most vulnerable, where 71.1% experience food insecurity [30]. As contact tracing requires a physical presence test, the follow-up of contacts can limit economic activities, and therefore make it difficult for some contacts to remain compliant for the full duration of the contact tracing window. This finding aligns with the community-based containment strategy implemented in DRC, which also evidenced a positive effect of food support on contact compliance [21].

Health areas with two or more teams were more likely to complete the 21-day follow-up. This is presumably due to better supervision and support of the contact tracing teams, and echoes findings from a previous study examining the West Africa epidemic [18]. However, it is important to note that the composition of the contact tracing teams—in addition to their number—should be considered. Multidisciplinary teams are often advised, so that visits to contacts can be limited to one or two visits per day. For example, the Ugandan authorities recently decided to send a single multidisciplinary team to provide an integrated response to Sudan Virus Disease hotspots, rather than sending multiple separate teams to operate in silos [31]. This approach provides an opportunity to present the community with a single point of contact from which a relationship may be built between the people responding to the outbreak and those affected by it. This, in turn, may allow for mutual understanding and the building of trust, thus enabling responders and community members to work together as partners thereby better containing the outbreak.

There was a strong association between whether individuals had been vaccinated for EVD and contact tracing completeness. This agrees with a recent study in the DRC, where vaccination uptake was demonstrated to be significantly higher among contacts who accepted community isolation (i.e., those who voluntarily self-quarantined at a dedicated site and were regularly monitored over the 21-day incubation period) [21]. This finding is likely because those accepting vaccination were already prone to wider acceptance of the milieu of public health interventions that comprised the overall EVD response. In other words, it is not vaccination that improved contact tracing completeness per se, but rather that those accepting vaccination were more accepting of the response and therefore contact tracing. While intuitive, this nevertheless has important implications when conducting contact tracing in future outbreaks, in that it may be prudent to commit more resources to tracing individuals who indicate reticence in this way. It also highlights the critical role of effective risk communication in public health interventions [32].

Whether these factors are shared in other countries at other times cannot be predicted using the results of this study—indeed, these findings corroborate the conclusions of some prior reports but disagree with others. What is clear is that certain demographic factors, and certain factors related to the organization of a response, can have significant effect on the probability of successful contact tracing. Understanding and considering these (perhaps context-specific) factors and using this knowledge to adjust resource allocation and contact tracing strategies, is evidently crucial to improve the completeness of contact tracing in future epidemics.

## Conclusion

This study confirms that the completeness of 21-day follow-up in the 2021 EVD outbreak in Guinea was influenced in a statistically significant way by factors related to: the socio-demographic characteristics of contacts (such as age, gender, and education); and factors related to the organization of the response (including the overall number of contact tracing teams for a given area, and the support provided to contacts during their follow-up period such as food donations). This has important implications for how contact tracing during outbreaks of viral hemorrhagic fevers and other infectious diseases in similar contexts is designed and organized. Examining and understanding these factors—and adapting contact tracing accordingly, especially when related to organizational factors within the control of the response—may be key to saving lives and shortening life-threatening epidemics.

## Data Availability

Data are available on reasonable request.

## Abbreviations

CI: Confidence interval
EVD: Ebola virus disease
OR: Odds ratio
WHO: World Health Organization

## References

[1] Keita AK, Koundouno FR, Faye M, et al. Resurgence of Ebola virus in 2021 in Guinea suggests a new paradigm for outbreaks. Nature. 2021;597(7877):539–43. 10.1038/s41586-021-03901-9. 34526718 10.1038/s41586-021-03901-9

[2] Ohimain EI, Silas-Olu D. The 2013–2016 Ebola virus disease outbreak in West Africa. Curr Opin Pharmacol. 2021;60:360–5. 34537503 10.1016/j.coph.2021.08.002

[3] Jacob ST, Crozier I, Fischer WA, et al. Ebola virus disease. Nat Rev Dis Prim. 2020. 10.1038/s41572-020-0147-3. 32080199 10.1038/s41572-020-0147-3 PMC7223853

[4] Subissi L, Keita M, Mesfin S, et al. Ebola virus transmission caused by persistently infected survivors of the 2014–2016 outbreak in West Africa. J Infect Dis. 2018;218(Suppl 5):2014–8. 10.1093/infdis/jiy280 PMC6249578 29920602

[5] Mbala-Kingebeni P, Pratt C, Mutafali-Ruffin M, et al. Ebola virus transmission initiated by relapse of systemic Ebola virus disease. N Engl J Med. 2021;384(13):1240–7. 33789012 10.1056/NEJMoa2024670 PMC7888312

[6] World Health Organization (2014) Contact tracing during an outbreak of Ebola virus disease. [cited 2024 Jan 10]. https://apps.who.int/iris/bitstream/handle/10665/159040/9789290232575.pdf

[7] Saurabh S, Prateek S. Role of contact tracing in containing the 2014 Ebola outbreak: a review. Afr Health Sci. 2017;17(1):225–36. 29026397 10.4314/ahs.v17i1.28 PMC5636234

[8] Ilunga Kalenga O, Moeti M, Sparrow A, Nguyen V-K, Lucey D, Ghebreyesus TA. The ongoing Ebola epidemic in the Democratic Republic of Congo, 2018–2019. N Engl J Med. 2019;381(4):373–83. 31141654 10.1056/NEJMsr1904253

[9] Aruna A, Mbala P, Minikulu L, et al. Ebola virus disease outbreak—Democratic Republic of the Congo, August 2018–November 2019. MMWR Morb Mortal Wkly Rep. 2019;68(50):1162–5. 31856146 10.15585/mmwr.mm6850a3 PMC6936163

[10] Keita M, Talisuna A, Chamla D, et al. Investing in preparedness for rapid detection and control of epidemics: analysis of health system reforms and their effect on 2021 Ebola virus disease epidemic response in Guinea. BMJ Glob Health. 2023;8(1):e010984. 36599498 10.1136/bmjgh-2022-010984 PMC9815045

[11] Polonsky JA, Böhning D, Keita M, et al. Novel use of capture-recapture methods to estimate completeness of contact tracing during an Ebola outbreak, Democratic Republic of the Congo, 2018–2020. Emerg Infect Dis. 2021;27(12):3063–72. 34808076 10.3201/eid2712.204958 PMC8632194

[12] Braithwaite I, Callender T, Bullock M, Aldridge RW. Automated and partly automated contact tracing: a systematic review to inform the control of COVID-19. Lancet Digit Health. 2020;2(11):e607–21. 32839755 10.1016/S2589-7500(20)30184-9 PMC7438082

[13] Institut National de la Statistique de Guinée. Troisième Recensement Général de la Population et de l’Habitation (RGPH3) [Internet]. [cited 2023 Feb 21]. https://www.stat-guinee.org/images/Documents/Publications/INS/rapports_enquetes/RGPH3/RGPH3_etat_structure.pdf

[14] R Core Team (2014) A language and environment for statistical computing. R Foundation for Statistical Computing. Vienna, Austria. http://www.r-project.org/

[15] Centers for Disease Control and Prevention, National Center for Emerging and Zoonotic Infectious Diseases (NCEZID), Division of High-Consequence Pathogens and Pathology (DHCPP) VSPB (VSPB). History of Ebola Virus Disease (EVD) Outbreaks. [cited 2023 Feb 25]. https://www.cdc.gov/vhf/ebola/history/chronology.html

[16] Hossain AD, Jarolimova J, Elnaiem A, Huang CX, Richterman A, Ivers LC. Effectiveness of contact tracing in the control of infectious diseases: a systematic review. Lancet Public Health. 2022;7:e259–73. 35180434 10.1016/S2468-2667(22)00001-9 PMC8847088

[17] Longley JL, Danquah LO, Massa MS, Ross DA, Weiss HA. The impact of case and contact characteristics on contact tracing during the West Africa Ebola epidemic. J Infect. 2021;83(4):496–522. 34245765 10.1016/j.jinf.2021.07.002

[18] Reques L, Bolibar I, Chazelle E, et al. Evaluation of contact tracing activities during the Ebola virus disease outbreak in Guinea, 2015. Int Health. 2017;9(2):131–3. 28338749 10.1093/inthealth/ihx004

[19] Senga M, Koi A, Moses L, et al. Contact tracing performance during the Ebola virus disease outbreak in Kenema district, Sierra Leone. Philos Trans R Soc B Biol Sci. 2017;372(1721):20160300. 10.1098/rstb.2016.0300 PMC5394638 28396471

[20] Swanson KC, Altare C, Wesseh CS, et al. Contact tracing performance during the Ebola epidemic in Liberia, 2014–2015. PLoS Negl Trop Dis. 2018;12(9):e0006762. 30208032 10.1371/journal.pntd.0006762 PMC6152989

[21] Keita M, Polonsky JA, Ahuka-Mundeke S, et al. A community-based contact isolation strategy to reduce the spread of Ebola virus disease: an analysis of the 2018–2020 outbreak in the Democratic Republic of the Congo. BMJ Glob Health. 2023;8(6):e011907. 37263672 10.1136/bmjgh-2023-011907 PMC10254818

[22] Dixon MG, Taylor MM, Dee J, et al. Contact tracing activities during the Ebola virus disease epidemic in Kindia and Faranah, Guinea, 2014. Emerg Infect Dis. 2015;21(11):2022. 26488116 10.3201//eid2111.150684 PMC4622253

[23] Henao-Restrepo AM, Camacho A, Longini IM, et al. Efficacy and effectiveness of an rVSV-vectored vaccine in preventing Ebola virus disease: final results from the Guinea ring vaccination, open-label, cluster-randomised trial (Ebola Ça Suffit!). Lancet. 2017;389(10068):505–18. 28017403 10.1016/S0140-6736(16)32621-6 PMC5364328

[24] World Health Organization. Helping Guinean communities fight Ebola. Wkly Epidemiol Rec Relevé épidémiologique hebdomadaire. 2015;90(28):362–4. 26164868

[25] Fairhead J. Understanding social resistance to the Ebola response in the forest region of the Republic of Guinea: an anthropological perspective. Afr Stud Rev. 2016;59(3):7–31.

[26] Fawole OI, Bamiselu OF, Adewuyi PA, Nguku PM. Gender dimensions to the Ebola outbreak in Nigeria. Ann Afr Med. 2016;15(1):7. 26857931 10.4103/1596-3519.172554 PMC5452690

[27] Tenkorang EY. Effect of knowledge and perceptions of risks on Ebola-preventive behaviours in Ghana. Int Health. 2018;10(3):202–10. 10.1093/inthealth/ihy009. 29506203 10.1093/inthealth/ihy009

[28] Ross CE, Wu C. The links between education and health. Am Sociol Rev. 1995;60:719–45.

[29] Schone BS, Weinick RM. Health-related behaviors and the benefits of marriage for elderly persons. Gerontologist. 1998;38(5):618–27. 9803650 10.1093/geront/38.5.618

[30] Ly MS, Dou X. Strategies to resolve food insecurity in guinea international cooperation approaches (availability: production, distribution, and exchange of food): a case study in Guinea. Int J Econ Financ. 2020;12(11):1–59.

[31] Sprecher A. Understanding the key to outbreak control—Sudan virus disease in Uganda. N Engl J Med. 2022;387(26):2393–5. 36383468 10.1056/NEJMp2213975

[32] Scholz J, Wetzker W, Licht A, et al. The role of risk communication in public health interventions. An analysis of risk communication for a community quarantine in Germany to curb the SARS-CoV-2 pandemic. PLoS ONE. 2021;16(8):e0256113. 34388211 10.1371/journal.pone.0256113 PMC8362954

